# Shining a light on UV-fluorescent floral nectar after 50 years

**DOI:** 10.1038/s41598-024-62626-7

**Published:** 2024-05-25

**Authors:** Brandi Zenchyzen, John H. Acorn, Kian Merkosky, Jocelyn C. Hall

**Affiliations:** 1 Department of Biological Sciences, University of Alberta, Edmonton, AB T6G 2E9 Canada; 2 Department of Renewable Resources, University of Alberta, Edmonton, AB T6G 2E9 Canada

**Keywords:** Plant sciences, Plant signalling

## Abstract

Nature is aglow with numerous captivating examples of UV-fluorescence in the animal kingdom. Despite a putative role as a visual signal, exploration of UV-fluorescence in plants and its role in plant-animal interactions is lagging in comparison. Almost 50 years ago, UV-fluorescence of floral nectar, a crucial reward for pollinators, was reported for 23 flowering plant species. Since this intriguing discovery, UV-fluorescent nectar has only seldom been addressed in the scientific literature and has not been scrutinized in a phylogenetic or ecological context. Here, we report the prevalence of vibrant UV-fluorescent floral nectar across the family Cleomaceae, including the first photographic documentation in vivo colour for flowering plants. Though Cleomaceae flowers are morphologically diverse varying in colour, nectary prominence, and nectar volume, UV-fluorescent floral nectar may be a ubiquitous characteristic of the family. Fluorescence spectra show that the identity and number of fluorescent compounds in floral nectar may differ among Cleomaceae species. As Cleomaceae pollinators range from insects to bats and birds, we suggest that the UV-fluorescent floral nectar not only functions as a visual cue for the diurnal pollinators but also for the nocturnal/crepuscular pollinators in low light settings.

## Introduction

From vibrant colours to striking patterns, flowering plants display an astonishing array of characteristics that act as visual signals for pollinators^1^. The innate and learned preferences of pollinators to these suites of visual cues encourage visitation^2,3^, with flowering plants commonly offering rewards such as nectar in exchange for pollen transfer. Given the intricate ties among pollinators and floral displays, flower colour research is key to shedding light on plant-pollinator interactions and the evolution and diversity of floral form. Ultraviolet (UV)-fluorescence, a floral feature that may function as a visual signal for pollinator attraction, represents a significant gap in our knowledge of flower colour.

UV-fluorescence is a type of luminescence in which UV radiation is absorbed and longer wavelength light is emitted. Unlike the well-known UV nectar guides (i.e., patterns of UV-absorbance and -reflectance) that can only be perceived by animals with UV-receptors (e.g., insects and birds)^1,4^, the lower energy light emitted via UV-fluorescence can occur in the spectral range visible to humans (and many other animals)^1,5^. Further, UV-fluorescence has not been studied as extensively as UV nectar guides^1,6–8^. However, UV-fluorescence is of growing interest with many recent discoveries of this phenomenon across the animal kingdom (e.g., the hair of nocturnal springhare and flying squirrels^9,10^, skin of salamanders and catsharks^11,12^, and the bones of toadlets and chameleons^13,14^). Behavioural studies within the animal kingdom suggest that UV-fluorescence may be more than a coincidental by-product of chemical structure^15–17^. For example, female jumping spiders (*Cosmophasis umbratica*, Salticidae) have appendages that fluoresce bright green under UV radiation; in the absence of UV radiation, male jumping spiders do not perform typical courtship behaviour with non-fluorescing females^17^. Similarly, budgerigars (*Melopsittacus undulatus*, Psittacidae) have UV-fluorescent yellow plumage on their crown and cheeks; both sexes prefer budgerigars of the opposite sex with fluorescent plumage over those with masked fluorescence (i.e., concealed with UV-absorbing chemicals)^15^. Despite numerous captivating examples and a putative function as a visual signal in animals, the prevalence and significance of UV-fluorescence across flowering plants has scarcely been investigated.

Nearly 50 years ago, Thorp et al.^18^ were among the first to report the brilliant UV-fluorescence of nectar in flowering plants and suggested that this phenomenon functions as a visual signal for bees. Out of the 102 flowering plant species examined, 23 had nectar that fluoresced yellow to blue with varying degrees of intensity and the majority pollinated by bees^18^. Apart from nectar fluorophore (i.e., fluorescent molecule) identification for three species^19–21^, our understanding of UV-fluorescent nectar has seen limited progress since its discovery. Suggestions that nectar fluorescence could be used as an indicator of secretory cells in vivo^22,23^ and conceptual arguments about its ecological importance^24,25^ have not significantly enriched our fundamental knowledge of UV-fluorescent nectar. Yet, the presence of UV-fluorescence was recently reported in the prey traps of several carnivorous plant species and in the anthers and pollen of numerous flowering plants species^26–28^. Like the UV-fluorescent animal studies, behavioural experiments with UV-fluorescent prey traps and an anther/pollen fluorophore show that this phenomenon plays a role in animal attraction^26,27^. Pitcher plants (*Nepenthes khasiana*, Nepenthaceae) with masked fluorescence catch significantly less insect prey^26^ and bees are attracted to filter paper containing a fluorescent compound identified from anthers and pollen^27^. Though UV-fluorescence presumably contributes to the array of visual cues involved in pollinator attraction, further exploration is needed to determine the prevalence of UV-fluorescent nectar across flowering plants, characterize its molecular basis, and to establish a link to pollinator interactions.

**Figure 1:**
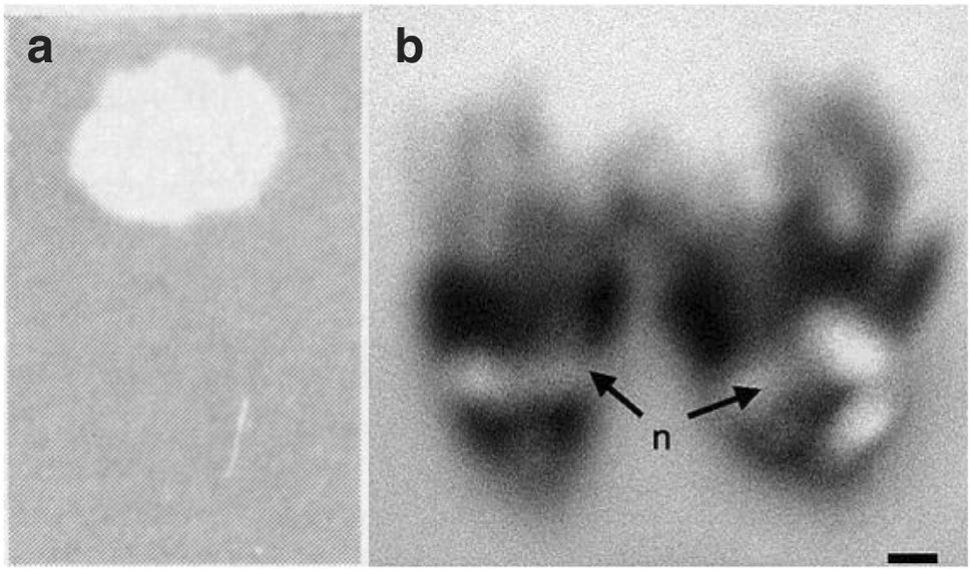
UV-fluorescent floral nectar of *Prunus* species. (**a**) A spot of *P. amygdalus* nectar on filter paper under long- and short-wavelength UV radiation. From Thorp et al.^18^. Reprinted with permission from AAAS. (**b**) A *P*. *persica* flower with nectar under UV radiation. From Radice and Galati^29^. Reprinted with permission from SNCSC. n, nectar. Scale bar, 2.5 mm (**b**).

Though UV-fluorescence in nature is a fascinating phenomenon to observe, to the best of our knowledge, there are only four published photographs of UV-fluorescent nectar^18,29,30^ (Fig. 1). Unfortunately, the photographs are monochromatic, captured ex vivo or marred by blurriness, and fail to ignite curiosity parallel to that of UV-fluorescence research in the animal kingdom. Here, we present the first in vivo colour images of UV-fluorescent nectar in flowering plants. We show UV-fluorescent nectar across Cleomaceae, a relatively small family with morphologically diverse flowers and a broad range of diurnal and nocturnal/crepuscular pollinators^31,32^. Moreover, we discuss the current state of flower florescence research, including the potential phylogenetic and ecological implications of UV-fluorescent floral nectar.

## Methods

### Plant material

The following 10 Cleomaceae and one Brassicaceae species were grown from seed in professional growing mix (Sun Gro Horticulture, Agawam, MA, USA): *Arivela viscosa* (L.) Raf. accession 815 from Hortus Botanicus, *Cleome amblyocarpa* Barratte & Murb. accession 151485 from Royal Botanic Gardens Kew, *Cleome violacea* L. accession 813 from Hortus Botanicus, *Gynandropsis gynandra* (L.) Briq. accession TOT8917 kindly provided by M. Eric Schranz, *Melidiscus giganteus* (L.) Raf. accession 814 from Hortus Botanicus, *Polanisia dodecandra* (L.) DC. accession 68456 from B & T World Seeds, *Sieruela hirta* (Klotzsch) Roalson & J. C. Hall accession 74520 from B & T World Seeds, *Sieruela monophylla* (L.) Roalson & J. C. Hall accession 353278 from Royal Botanic Gardens Kew, *Sieruela rutidosperma* (DC.) Roalson & J. C. Hall accession 512496 from B & T World Seeds, *Tarenaya houtteana* (Schltdl.) Soares Neto & Roalson (see Neto et al*.*^33^ for the recent taxonomic revision) accession FL2400 from West Coast Seeds, and *Brassica rapa* L. Wisconsin Fast Plants cultivar. Cleomaceae species were grown in CMP3244 growth chambers (Environmental Growth Chambers, Chagrin Falls, OH, USA) set to a 28 °C 12 h light and 22 °C 12 h dark regime and *B. rapa* was grown in a greenhouse (Greenhouse Complex, Department of Biological Sciences, University of Alberta). Jocelyn C. Hall and Brandi Zenchyzen formally identified the plant material. Voucher specimens were deposited in the University of Alberta Vascular Plant Herbarium (ALTA; Supplementary Table S1).

### Nectar volume measurements

Nectar was extracted from flowers with microcapillary tubes (0.4 mm i.d., 75 mm length; Drummond Scientific, Broomall, PA, USA) between 10:00 and 12:00. The height of nectar drawn into the microcapillary tube was measured to the nearest 0.5 mm and used to calculate nectar volume. For species with nectar too viscous to draw into a microcapillary tube (i.e., *M. giganteus* and *T. houtteana*) the following protocol was used: the flower was removed from the plant at the base of the pedicel, water was added to the nectar with a pipette, the pipette tip was used to manually swirl the nectar solution without aspirating, the nectar solution was extracted from the nectary with microcapillary tubes, and the amount of added water was subtracted from the total nectar solution volume. For each Cleomaceae species, nectar was collected from a minimum of three plants (i.e., biological replicates) and 1–30 flowers per plant. The five species with the highest nectar volumes were selected for in vivo photography and fluorescence spectra analysis.

### Photography and fluorescence spectroscopy

Floral nectar was extracted with microcapillary tubes or a pipette between 10:00 and 12:00 and pooled from multiple flowers and plants for each species. Samples were stored at 4 °C between extractions and until photography or fluorescence spectroscopy analysis. Flowering plants and floral nectar in microcapillary tubes were photographed in a dark room against a black background while illuminated with an iLED gooseneck illuminator (i.e., white light; Laxco, Mill Creek, WA, USA) or C8 Convoy 365 nm UV flashlights (Yooperlites, Brimley, MI, USA). Photographs were captured using a D80 DLSR camera (Nikon, Tokyo, Japan) with an AF Micro-NIKKOR 60 mm f/2.8 D or AF Micro-NIKKOR 105 mm f/2.8 AI-S lens (Nikon) or a D500 DLSR camera (Nikon) with AF-S Teleconverter TC-14 III and AF-S Micro-NIKKOR 105 mm f/2.8 IF-ED lenses (Nikon). Photoshop (Adobe; San Jose, CA, USA) was used to darken the black backgrounds. As Brassicaceae is sister family to Cleomaceae, the nectar of *B. rapa* was photographed and compared to that of the Cleomaceae species.

Fluorescence excitation and emission spectra were measured using quartz cuvettes and a PTI QM-8075-11 spectrofluorometer (Horiba, Kyoto, Japan) for pooled nectar samples, each totaling approximately 120 µL, and a genistein standard (Toronto Research Chemicals, Toronto, ON, Canada) in ethanol. Highly viscous samples were diluted with ultrapure water for ease of transfer. Cleomaceae nectar fluorescence spectra were compared to that of genistein and hydroxycinnamate derivatives (reported by Mori et al.^27^).

### Literature review

A literature review was conducted using Google Scholar to determine the phylogenetic distribution and possible ecological implications of UV-fluorescent nectar. As Thorp et al.^18^ is the key article that brought awareness to UV-fluorescent nectar, scientific literature citing this work was examined for mentions of additional species exhibiting this phenomenon and evaluations of its function. Further, we extended our review by searching for scientific literature containing the terms “ultraviolet”, “fluorescent”, and “nectar”. Studies that examined nectar for fluorescence after extraction from a pollinator or introduction of a solvent or stain were excluded due to the possibility of nectar modification. Examples of UV-fluorescence in other plant features were identified through review of the abovementioned literature. All methods were performed in compliance with the relevant guidelines and legislation.

## Results

**Figure 2:**
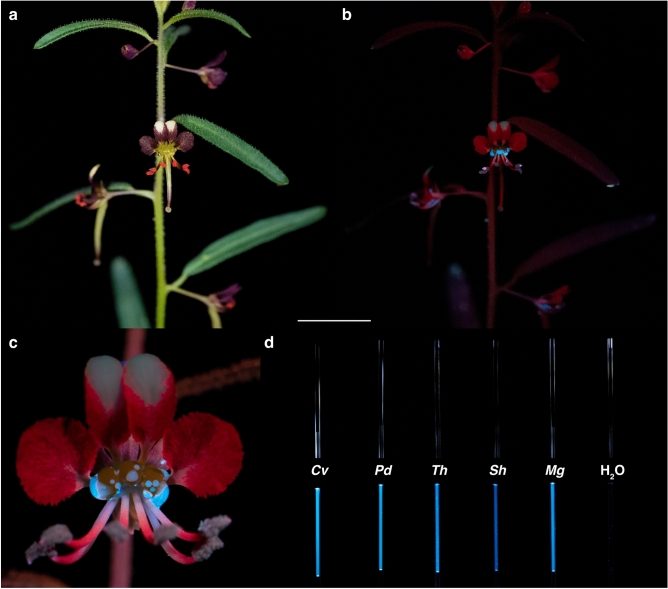
UV-fluorescent floral nectar of *Cleome violacea* and other Cleomaceae species. (**a**, **b**) *Cleome violacea* under white light (**a**) and UV-A radiation (**b**). (**c**) Close up of *C. violacea* flower under UV-A radiation. (**d**) Nectar of five Cleomaceae species and water in microcapillary tubes under white light (top) and UV-A radiation (bottom). *Cv*, *Cleome violacea*; *Pd*, *Polanisia dodecandra*; *Th*, *Tarenaya houtteana*; *Sh*, *Sieruela hirta*; *Mg*, *Melidiscus giganteus*. Scale bar, 1 cm (**a**, **b**).

For the nine Cleomaceae species examined here, nectar is secreted by receptacular nectaries located between the perianth and stamens, or perianth and androgynophore (i.e., stalk-like structure subtending the reproductive organs; e.g., *G. gynandra*)^32,34^. The nectaries are diverse in form, ranging from adaxial protrusions or concavities to annular disks, and from inconspicuous to a prominent component of the flower^32^. The average nectar volume secreted varied from 0.01 to 19.73 µL with the highest average nectar volumes (> 0.5 µL) secreted by *M. giganteus*, *T. houtteana, P. dodecandra*, *S. hirta*, and *C. violacea* and lowest (< 0.5 µL) by *S. rutidosperma*, *C. amblyocarpa*, *S. monophylla*, and *A. viscosa* (in descending order; Table 1). Of note, Lunau et al*.*^35^ mentioned *S*. *monophylla* as an example of a species with a false nectary (i.e., a glossy surface mimicking nectar); however, we show that its nectary is functional. *Arivela viscosa* was excluded from subsequent fluorescence analyses due to insufficient nectar volumes. For the remaining nine Cleomaceae species, the floral nectar is colourless under white light but exhibits vibrant blue fluorescence when illuminated by UV-A radiation with peak intensity at 365 nm (Fig. 2).

**Table 1:** Mean (± s.d.) floral nectar volume of nine Cleomaceae species.

Species	Nectar volume (µL)
*Arivela viscosa*	0.01 ± 0.02 (*N* = 58)
*Cleome amblyocarpa*	0.19 ± 0.10 (*N* = 93)
*Cleome violacea*	0.55 ± 0.20 (*N* = 130)
*Gynandropsis gynandra*	0.44 ± 0.25 (*N* = 78)
*Melidiscus giganteus*	19.73 ± 11.00 (*N* = 42)
*Polanisia dodecandra*	2.78 ± 0.59 (*N* = 90)
*Sieruela hirta*	2.12 ± 0.57 (*N* = 81)
*Sieruela monophylla*	0.07 ± 0.08 (*N* = 67)
*Sieruela rutidosperma*	0.31 ± 0.16 (*N* = 100)
*Tarenaya houtteana*	6.20 ± 2.07 (*N* = 68)

**Figure 3:**
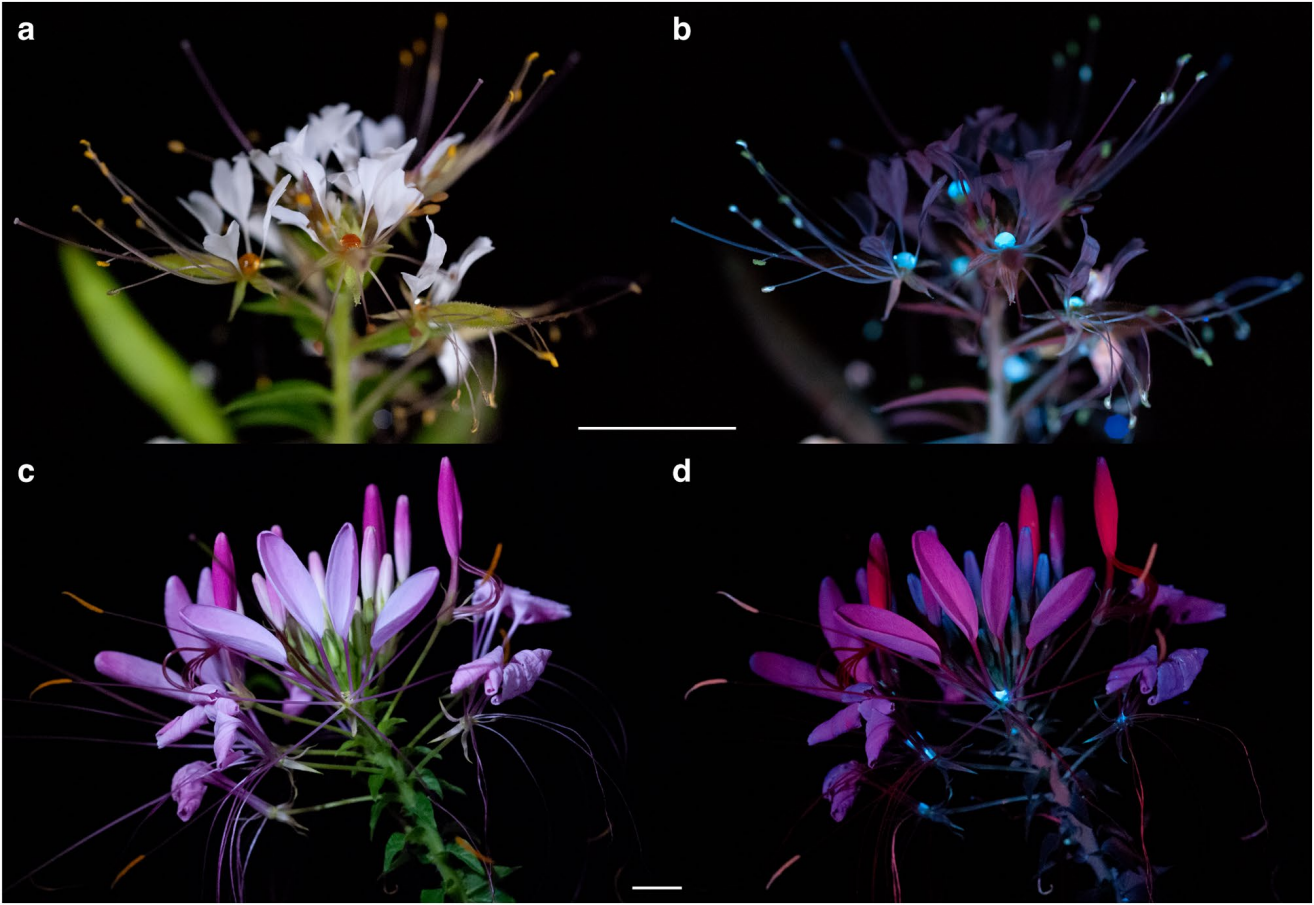
UV-fluorescent floral nectar of *Polanisia dodecandra* and *Tarenaya houtteana*. (**a**, **b**) *Polanisia dodecandra* under white light (**a**) and UV-A radiation (**b**). (**c**, **d**) *Tarenaya houtteana* under white light (**c**) and UV-A radiation (**d**). Scale bars, 1 cm.

With nectaries and nectar that are not obscured by the perianth or stamens, *C. violacea*, *P. dodecandra*, and *T. houtteana* have the most visually striking examples of UV-fluorescent nectar. Under white light, the nectar of *C*. *violacea* is challenging to discern from the green three-lobed nectary (Fig. 2a). Yet, when excited with UV-A radiation, the vividly fluorescent nectar droplets are easily distinguished from the nectary and contrast the less intense red fluorescence of chlorophyll^36^ (Fig. 2b,c). Similarly, under white light, the nectar of *P. dodecandra* and *T. houtteana* accumulates on top of an orange cup-shaped nectary and light green ‘V’-shaped nectary, respectively (Fig. 3a,c). Under UV-A radiation, the nectar of both species intensely fluoresces (Figs. 2d, 3b,d). In addition, the vasculature within the petals of *P. dodecandra* fluoresces blue and the petals of *T. houtteana* fluoresce bright pink.

**Figure 4:**
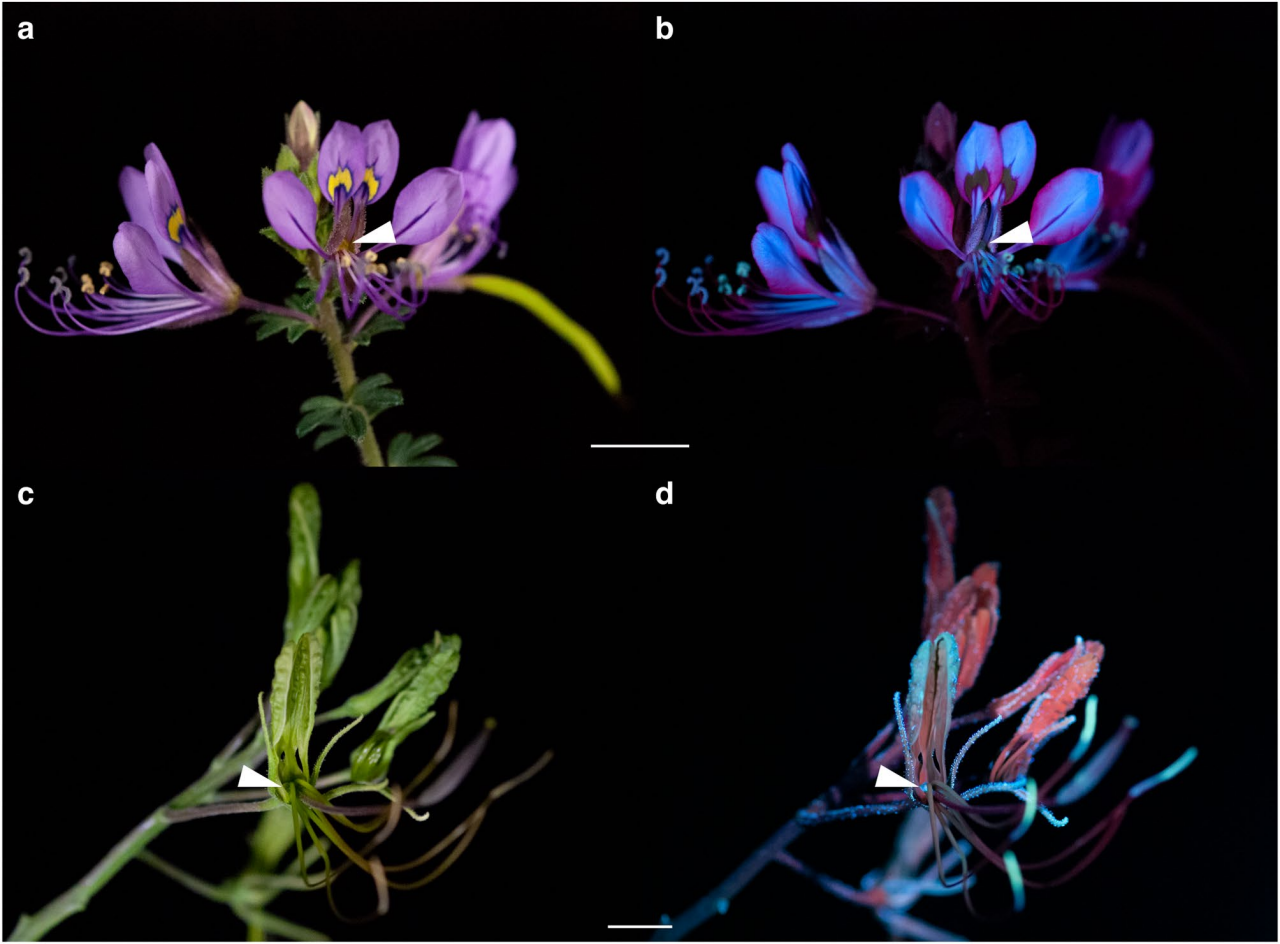
UV-fluorescent floral nectar of *Sieruela hirta* and *Melidiscus giganteus*. (**a**, **b**) *Sieruela hirta* under white light (**a**) and UV-A radiation (**b**). (**c**, **d**) *Melidiscus giganteus* under white light (**c**) and UV-A radiation (**d**). Arrowheads are pointing to the partially exposed nectar. Scale bars, 1 cm.

Cleomaceae species with nectaries and nectar partially or entirely concealed by the perianth and stamens include *C. amblyocarpa*, *M. giganteus*, *S. hirta*, *S. monophylla*, and *S. rutidosperma*. For instance, the perianth of *S. hirta* and *M. giganteus* partially obscure the adaxially secreted nectar (Fig. 4a,c). Though both species secrete UV-fluorescent nectar (Fig. 2d), the blue fluorescence of the nectar does not appear as vibrant against the intense fluorescence of the other floral structures (Fig. 4b,c). As *C. amblyocarpa*, *G. gynandra*, *S. monophylla*, and *S. rutidosperma* have obscured nectaries and/or low nectar volumes (< 0.5 µL), nectar was pooled from multiple flowers and observed in microcapillary tubes. Whether exposed or obscured, the nectar of the nine Cleomaceae species fluoresces blue under UV-A radiation (Fig. 2d, Supplementary Fig. S1). The intensity of fluorescence varies between and within genera and can differ within species (Supplementary Fig. S1). Of note, the pollen fluorescence can also be rather vivid though the nectar fluorescence often steals the show (i.e., *P. dodecandra*) (Fig. 3b). Like the Cleomaceae taxa, *B. rapa* has receptacular nectaries between the perianth and stamens and UV-fluorescent nectar (Supplementary Fig. S1).

**Figure 5:**
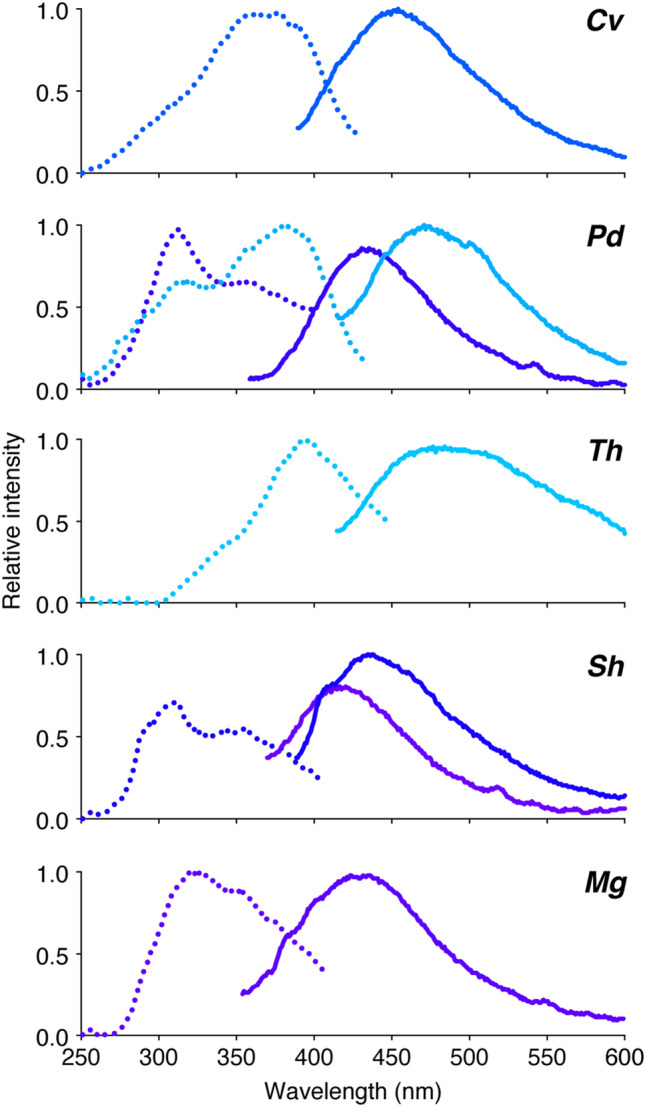
Fluorescence spectra for the floral nectar of five Cleomaceae species. Excitation spectra, dotted lines; emission spectra, solid lines. Colours correspond to the human perceived colours for the emission spectra maximum wavelengths. *Cv*, *Cleome violacea*; *Pd*, *Polanisia dodecandra*; *Th*, *Tarenaya houtteana*; *Sh*, *Sieruela hirta*; *Mg*, *Melidiscus giganteus*.

For the five species that secreted the greatest volumes of nectar, the nectar fluorescence excitation maxima ranged from 309 to 396 nm and the emission maxima varied from 416 to 476 nm (Fig. 5, Supplementary Table S2). The nectar fluorescence spectra of *C. violacea*, *M. giganteus*, and *T. houtteana* each had one set of emission and excitation peaks, while *P. dodecandra* and *S. hirta* had two sets of peaks. No two species had the same nectar fluorescence spectra. The Cleomaceae nectar fluorescence spectra maxima did not correspond to that of genistein (Supplementary Table S2), which fluoresced green instead of blue, but more closely resembled the fluorescence spectra of hydroxycinnamate derivatives^27^.

## Discussion

To our knowledge, our study is the first report of widespread UV-fluorescent floral nectar across a flowering plant family and the only photographic documentation of this phenomenon in vivo colour. Adding to the numerous reports of diverse animals exhibiting UV-fluorescence, we show that the floral nectar of nine representatives from six Cleomaceae genera fluoresces vibrant blue when illuminated with UV radiation.

### Evolution of UV-fluorescent nectar

**Figure 6:**
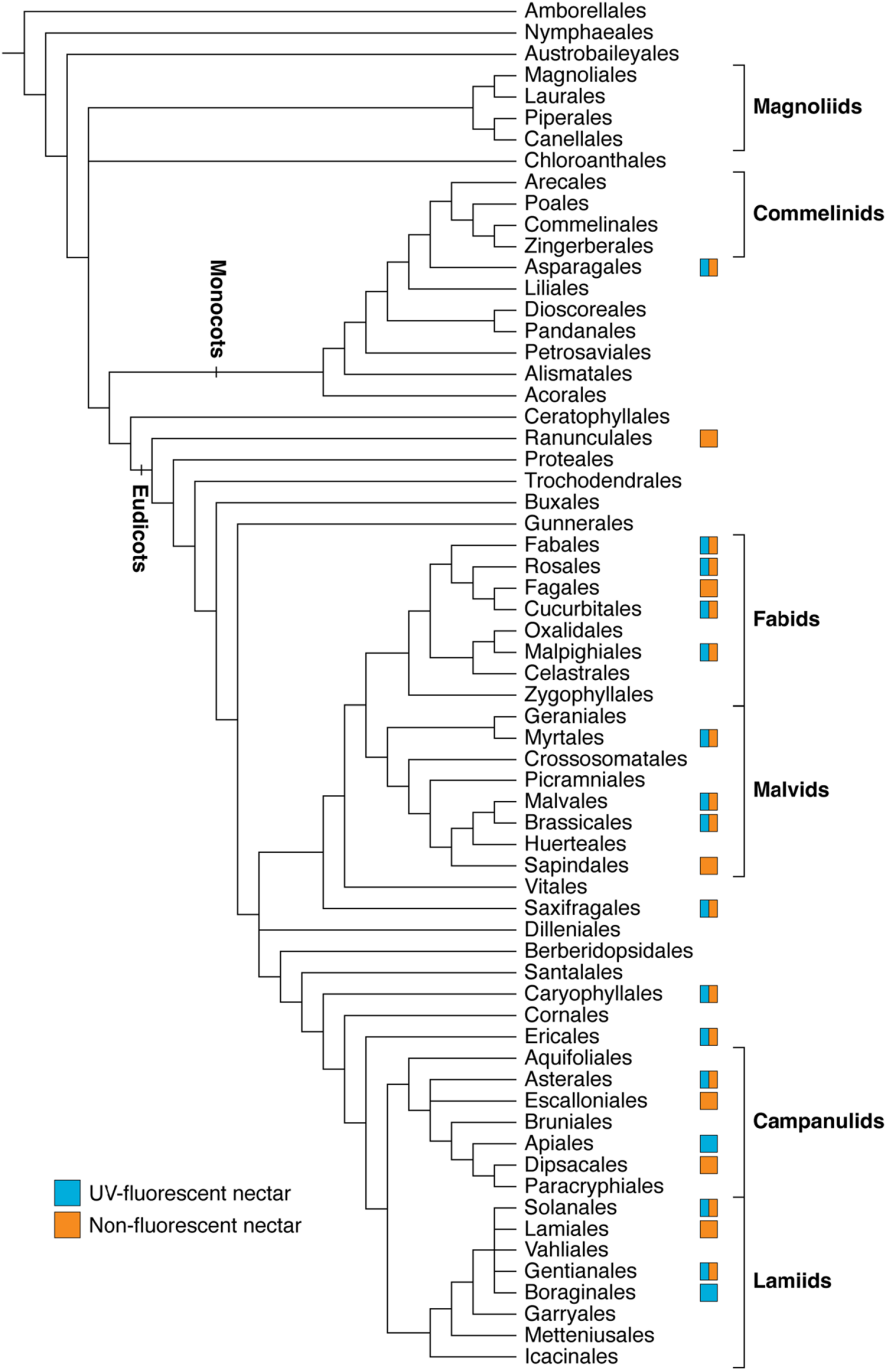
Phylogenetic distribution of UV-fluorescent and non-fluorescent floral nectar across flowering plants. A box is present for orders that have data for at least one species. The phylogeny is adapted from that of the Angiosperm Phylogeny Group^67^ and data is summarized from Thorp et al*.*^18^, Scogin^21^, Roshchina et al*.*^22^, Nakanishi^30^, Davis et al.^37^, and the present study.

Though there is limited evidence about its prevalence, the scattered distribution of UV-fluorescent nectar suggests that this phenomenon arose multiple times across flowering plants (Fig. 6). Including the taxa from our investigation, UV-fluorescent nectar has been documented for 41 species (19 families), predominantly belonging to the eudicots^18,21,22,30,37^ (Table 2). For most of these families, the occurrence of UV-fluorescent nectar varies within family, genus, or even species (Table 2; Supplementary Table S3). For instance, Thorp et al*.*^18^ noted the presence of UV-fluorescent nectar in five *Prunus* (Rosaceae) species and absence in four, including UV-fluorescent nectar in peach but not nectarine flowers (i.e., varieties of *P. persica*). The variability between closely related individuals suggests that the genetic mechanisms governing fluorophore biosynthesis are conserved but differentially expressed within lineages. While nectar fluorescence is variable in Brassicaceae (sister family to Cleomaceae), this phenomenon may be a unifying feature of Cleomaceae as all ten species investigated, which span multiple genera and clades, exhibit nectar fluorescence to varying degrees of intensity^18^ (Supplementary Fig. S1).

**Table 2:** Flowering plant species with UV-fluorescent floral nectar. Adapted from the data of Thorp et al*.* ^18^, Scogin^21^, Roshchina et al*.* ^22^, Nakanishi^30^, Davis et al.^37^, and the present study (indicated by an asterisks).

Group	Order	Family	Species
Monocots	Asparagales	Amaryllidaceae	*Allium cepa*
		Asparagaceae	*Muilla maritima*
		Orchidaceae	*Maxillaria anceps*
Eudicots	Fabales	Fabaceae	*Glycine max*
			*Robinia pseudoacacia*
	Rosales	Rosaceae	*Photinia serratifolia* (formerly *P. serrulata*)
			*Prunus amygdalus* (formerly *P. dulcis*)
			*Prunus ilicifolia* (formerly *P. ilicifolia* and *P. lyonii*)
			*Prunus lusitanica*
			*Prunus mume*
			*Prunus persica*
			*Prunus salicina*
	Cucurbitales	Cucurbitaceae	*Cucumis sativus*
	Malpighiales	Euphorbiaceae	*Euphorbia pulcherrima*
		Passifloraceae	*Passiflora caerulea*
	Myrtales	Onagraceae	*Ludwigia peploides*
	Malvales	Malvaceae	*Bombax ceiba* (formerly *B. malabaricum*)
			*Fremontodendron californicum*
			*Fremontodendron mexicanum*
			*Pseudobombax ellipticum*
	Brassicales	Cleomaceae	Cleome amblyocarpa*
			Cleome violacea*
			*Cleomella arborea* (formerly *Isomeris arborea*)
			Gynandropsis gynandra*
			Melidiscus giganteus*
			Polanisia dodecandra*
			Sieruela hirta*
			Sieruela monophylla*
			Sieruela rutidosperma*
			Tarenaya houtteana*
		Brassicaceae	Brassica rapa*
			*Crambe maritima*
	Saxifragales	Crassulaceae	*Kalanchoe daigremontiana* (formerly *Bryophyllum daigremontianum*)
	Caryophyllales	Polygonaceae	*Fagopyrum esculentum*
	Ericales	Balsaminaceae	*Impatiens balsamina*
	Asterales	Asteraceae	*Centaurea solstitialis*
			*Lasthenia californica* (formerly *L. chrysostoma*)
	Apiales	Apiaceae	*Daucus carota*
	Solanales	Convolvulaceae	*Convolvulus arvensis*
	Gentianales	Apocynaceae	*Hoya carnosa*
	Boraginales	Hydrophyllaceae	*Phacelia viscida*

With the possibility of multiple independent origins of UV-fluorescent nectar, the question arises: are distinct fluorophores responsible for nectar fluorescence throughout the flowering plant phylogeny? Like nectar, species with anthers and pollen that emit blue fluorescence under UV radiation are dispersed across flowering plants^27,28^. Mori et al*.*^27^ identified the anther and pollen fluorophores of five species in four different eudicot families as hydroxycinnamate derivatives and suggested their widespread distribution and shared biosynthetic pathway for taxa with fluorescent blue anthers and pollen. Alternatively, UV-fluorescent compounds can be unique to specific clades and thus act as taxonomically informative characters. For example, ester-linked ferulic acid exclusively occurs in the cell walls of the monophyletic clades commelinid monocots and core Caryophyllales^38,39^. When observed with UV-fluorescence microscopy, cell walls with ester-linked ferulic acid fluoresce blue when in water and green when in acid^38^. For the five Cleomaceae species examined, the distinct fluorescence spectra suggest that different compound(s) are responsible for the nectar fluorescence in each species. Further, the two sets of excitation and emission maxima for *P. dodecandra* and *S. hirta* indicate two fluorophores may be present in the nectar of these species. Additional research is needed to identify the nectar fluorophores of Cleomaceae species and determine if the fluorescent compounds are from a shared biosynthetic class.

Though often perceived as a simple sugar solution, nectar consists of a complex array of biomolecules and microorganisms (i.e., bacteria and fungi)^34,40,41^. Major constituents such as water, carbohydrates, and amino acids reward pollinators; proteins can tailor nectar chemistry for pollinators and prevent microbial infections; and secondary metabolites including scented and coloured compounds can contribute to pollinator attraction^40–44^. Since biomolecules, including large macromolecules such as proteins, can act as fluorophores and microorganisms can contain fluorophores^36^, the complexity of nectar poses a challenge for the identification of UV-fluorescent components. Yet, in response to the fascinating finding of Thorp et al*.*^18^, Scogin^19–21^ identified the UV-fluorescent compounds in the nectar of three Malvaceae species as a genistein-related isoflavone and its glucoside (*Fremontodendron californicum* and *F. mexicanum*) and a hydroxycoumarin (*Bombax ceiba*). Since this discovery, there have been several reports of a hydroxycinnamate derivative (i.e., chlorogenic acid; pollen/anther fluorophore^27^), genistein-related isoflavone and glucosides, and a hydroxycoumarin (i.e., aesculetin) in the nectar of diverse taxa, including two species with known UV-fluorescent nectar^18,45–50^ (*Robinia pseudoacacia*, Fabaceae and *Fagopyrum esculentum*, Polygonaceae; Supplementary Table S4). However, these studies did not examine the nectar samples for UV-fluorescence.

### Potential ecological function of fluorescent nectar

This seemingly prevalent phenomenon in Cleomaceae brings forth the question: does UV-fluorescent nectar serve as a visual cue for the array of Cleomaceae pollinators? The significance of UV-fluorescence for pollinator attraction has been debated. Thorp et al*.*^18^ posited that UV-fluorescent nectar functions as a visual signal for foraging bees. However, this hypothesis has been criticized due to concerns that the emitted fluorescence may be imperceptible to insects amid the reflected light^24,25^. While describing the colour of a flower may appear straightforward, providing an ecologically relevant description proves challenging as colour perception is dependent on the sensory and processing capabilities of the observer^1^. For instance, insects and birds have UV photoreceptors, while humans do not^1,4^. As a result, the perceived colour of a flower may drastically differ between humans and pollinators. Under sunlight, nectar fluorescence may not be conspicuous to the human eye, yet behavioural assays have shown that honeybees make fine colour discriminations and are attracted to a UV-fluorescent compound (i.e., chlorogenic acid)^27,51^. Further, honeybees have a blue photoreceptor maximally activated at 436 nm, lying within the range of Cleomaceae nectar fluorescence emission maxima (416–476 nm; Fig. 5, Supplementary Table S2), and an innate preference for blue colours^52,53^.

Unlike Thorp et al*.*^18^ that reported bees as the primary pollinators of taxa with UV-fluorescent nectar, Cleomaceae consists of both generalist species pollinated by a variety of insects and sometimes hummingbirds (e.g., *A. viscosa*, *Cleomella arborea*, *P. dodecandra*)^54–56^ and specialist species solely pollinated by bats (e.g., *M. giganteus* and *T. houtteana*)^57,58^. Though echolocation and olfaction play important roles in bat orientation and foraging^59,60^, all bat species have functional eyes^61^. For example, one of the bat pollinators of *T. houtteana* (i.e., *Glossophaga soricine*, Phyllostomidae) is colour-blind but able to perceive UV radiation as well as some human-visible light^61^. Further, Domingos-Melo et al.^62^ suggested that bats are attracted to the brightness of visible light reflecting off chiropterophilous (i.e., bat-pollinated) flowers at dusk. In addition to bats, nocturnal/crepuscular pollinators of Cleomaceae also include hawkmoths (e.g., *G. gynandra*)^63^. Investigations on animals show that UV-fluorescence is most prevalent and intense for nocturnal species and suggest that this phenomenon is an overlooked visual signal for nocturnal animals^9,10,64–66^. Likewise, a behavioural assay involving the UV-fluorescent pitcher plant *N. khasiana* revealed that UV-fluorescence may play a role in insect attraction in low light settings^26^. Prey capture of unmasked pitcher plants primarily occurred at night and masking of the fluorescent blue rim significantly reduced the capture of insect prey^26^. With evidence from the behavioral assays suggesting fluorescence aids in the attraction of both diurnal and nocturnal/crepuscular pollinators^26,27^, it is possible that fluorescent nectar in Cleomaceae not only acts as a visual signal for daytime pollinators but may also assist in the attraction of nocturnal/crepuscular pollinators during twilight. Alternatively, or in addition to a visual role, nectar fluorophores may impact pollinator health. For instance, high concentrations of chlorogenic acid significantly reduce infection by a gut pathogen in the bumblebee *Bombus impatiens* (Apidae)^68^.

Studies on the visual cues of flowers have predominately focused on pigmentation that is reflective in the visible range, or UV-absorptive or -reflective, while neglecting UV-fluorescence as a potential signal for pollinators. Yet, amid the recent wave of UV-fluorescence discoveries in the animal kingdom, UV-fluorescence has emerged as a budding area of research accompanied by many outstanding questions. Here, we add Cleomaceae floral nectar to the growing collection of UV-fluorescence findings in nature and suggest: (1) fluorescent blue nectar may be a taxonomically informative characteristic for Cleomaceae, (2) several compounds are responsible for nectar fluorescence across Cleomaceae, and (3) UV-fluorescent nectar not only serves as a visual signal for bees but may also function as a visual cue for nocturnal/crepuscular pollinators, such as bats and hawkmoths, in low light settings. Whether tied to pollinator interactions or merely an incidental consequence of chemical structure, we hope the visually striking UV-fluorescence of Cleomaceae floral nectar prompts further systematic, chemical, and ecological investigations into the UV-fluorescence of flowering plants.

### Supplementary Information

Supplementary Information.

## Data Availability

All data generated during this study are included in this article.
