# Supplementary material for: Shining a light on UV-fluorescent floral nectar after 50 years
Source: Sci Rep. 2024 May 25;14:11992. doi: 10.1038/s41598-024-62626-7 (PMC11128001; doi:10.1038/s41598-024-62626-7)
Supplement: Supplementary file 1 — Supplementary Information. [file 41598_2024_62626_MOESM1_ESM.docx]

## Supplementary Information

**Shining a light on UV-fluorescent floral nectar after 50 years**

Brandi Zenchyzen^1^*, John H. Acorn^2^, Kian Merkosky^1^ & Jocelyn C. Hall^1^

^1^Department of Biological Sciences, University of Alberta, Edmonton, Alberta T6G 2E9, Canada

^2^Department of Renewable Resources, University of Alberta, Edmonton, Alberta T6G 2E9, Canada

*For correspondence: zenchyze@ualberta.ca

**Supplementary Figure S1.** Floral nectar of nine Cleomaceae and one Brassicaceae species and water in microcapillary tubes under white light (top) and UV-A radiation (bottom). Nectar was collected and photographed with the same aperture and shutter speed on two separate occasions. (**a**, **b**) Nectar of all species excluding *Melidiscus giganteus* (**a**) and *Sieruela monophylla* (**b**). Abbreviations: *Ca*, *Cleome amblyocarpa*; *Cv*, *Cleome violacea*; *Gg*, *Gynandropsis gynandra*; *Mg*, *Melidiscus giganteus*; *Pd*, *Polanisia dodecandra*; *Sh*, *Sieruela hirta*; *Sm*, *Sieruela monophylla*; *Sr*, *Sieruela rutidosperma*; *Th*, *Tarenaya houtteana*; *Br*, *Brassica rapa*.

**
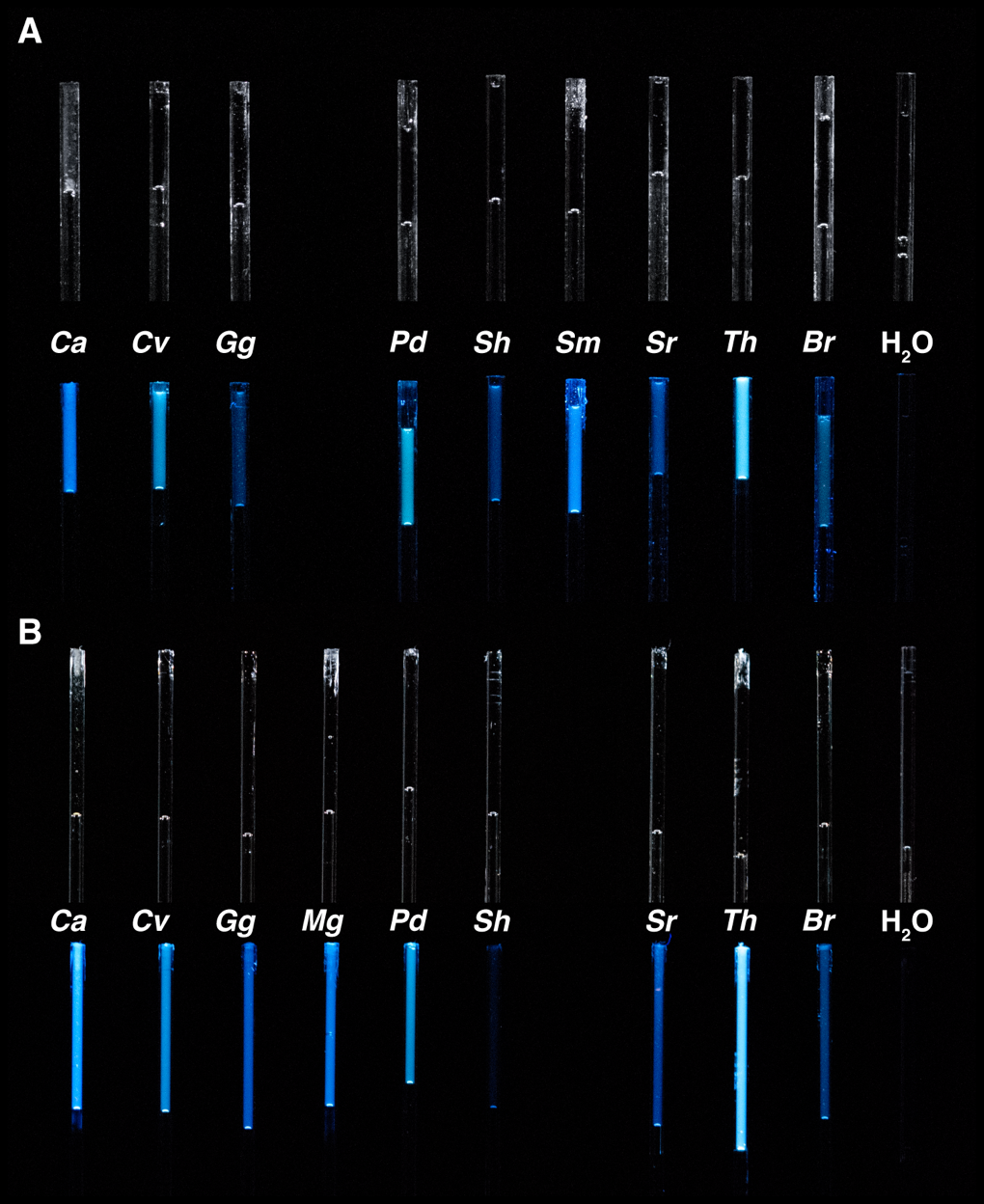
**

**Supplementary Table S1.** University of Alberta Vascular Plant Herbarium (ALTA) accession numbers for the 10 Cleomaceae species.

| Species | Herbarium Accession Number |
| --- | --- |
| *Arivela viscosa* | 143371 |
| *Cleome amblyocarpa* | 144822 |
| *Cleome violacea* | 144828 |
| *Gynandropsis gynandra* | 143369 |
| *Melidiscus giganteus* | 144833 |
| *Polanisia dodecandra* | 144836 |
| *Sieruela hirta* | 143370 |
| *Sieruela monophylla* | 144914 |
| *Sieruela rutidosperma* | 144838 |
| *Tarenaya hassleriana* | 144840 |

**Supplementary Table S2.** Maximum fluorescence excitation and emission wavelengths for the floral nectar of five Cleomaceae species and genistein standard.

| Species or standard | λ_ex_ (nm) | λ_em_ (nm) |
| --- | --- | --- |
| *Cleome violacea* | 358 | 454 |
| *Melidiscus giganteus* | 326 | 423 |
| *Polanisia dodecandra* | 312, 383 | 431, 471 |
| *Sieruela hirta* | 309, 354 | 416, 437 |
| *Tarenaya houtteana* | 396 | 476 |
| Genistein | 404 | 511 |

**Supplementary Table S3.** Flowering plant species with non-fluorescent floral nectar. Adapted from Thorp *et al*. [1] and Roshchina *et al.* [2], Scogin [3]. Bracketed numbers indicate the number of species from a genus. Of note, as numerous taxonomic changes have occurred in the past 50 years, without specific epithets it is impossible to ensure that the genera investigated by Thorp *et al.* [1] accurately reflect the present-day species.

| Group | Order | Family | Species |
| --- | --- | --- | --- |
| Monocots | Asparagales | Amaryllidaceae | *Allium* sp. |
|  |  | Asparagaceae | *Brodiaea* spp. (2) |
|  |  | Asphodelaceae | *Aloe* sp. |
|  |  | Melanthiaceae | *Zigadenus* sp. |
| Eudicots | Rununculales | Ranunculaceae | *Aquilegia* sp. |
|  |  |  | *Delphinium* sp. |
|  | Fabales | Fabaceae | *Cercis* sp. |
|  |  |  | *Medicago* sp. |
|  |  |  | *Melilotus* sp. |
|  |  |  | *Phaseolus* sp. |
|  |  |  | *Trifolium* spp. (3) |
|  | Rosales | Rosaceae | *Amelanchier* sp. |
|  |  |  | *Prunus* spp. (4) |
|  |  |  | *Pyracantha* sp. |
|  |  |  | *Pyrus* sp. |
|  |  |  | *Rubus* sp. |
|  |  |  | *Spiraea* sp. |
|  |  | Ulmaceae | *Ulmus* sp. |
|  | Fagales | Betulaceae | *Betula* sp. |
|  | Cucurbitales | Cucurbitaceae | *Cucurbita* sp. |
|  | Malpighiales | Euphorbiaceae | *Euphorbia* sp. |
|  |  |  | *Pedilanthus* sp. |
|  |  | Passifloraceae | *Passiflora* sp. |
|  |  | Salicaceae | *Populus* sp. |
|  |  |  | *Salix* sp. |
|  |  | Violaceae | *Viola* sp. |
|  | Myrtales | Myrtaceae | *Callistemon* sp. |
|  |  |  | *Eucalyptus* spp. (2) |
|  |  | Onagraceae | *Clarkia* sp. |
|  |  |  | *Fuchsia* sp. |
|  | Malvales | Malvaceae | *Ceiba acuminata* |
|  |  |  | *Durio zibethinus* |
|  |  |  | *Gossypium* sp. |
|  |  |  | *Ochroma pyramidale* |
|  |  |  | *Pachira aquatica* |
|  |  |  | *Pseudobombax grandiflorum* |
|  |  |  | *Tilia* spp. (2) |
|  | Brassicales | Brassicaceae | *Brassica* sp. |
|  |  |  | *Diplotaxis* sp. |
|  |  |  | *Nasturtium* sp. |
|  |  | Limnanthaceae | *Limnanthes* sp. |
|  | Sapindales | Sapindaceae | *Acer* spp. (3) |
|  |  |  | *Aesculus* sp. |
|  |  | Rutaceae | *Citrus* sp. |
|  |  |  | *Zanthoxylum* sp. (formerly *Xanthoxylum*) |
|  | Saxifragales | Crassulaceae | *Sedum* sp. |
|  |  | Grossulariaceae | *Ribes* sp. |
|  | Caryophyllales | Cactaceae | *Disocactus × hybridus* (formerly *Epiphyllum hybridum*) |
|  |  | Caryophyllaceae | *Dianthus* sp. |
|  | Ericales | Ericaceae | *Arctostaphylos* sp. |
|  |  |  | *Rhododendron* sp. |
|  |  | Polemoniaceae | *Navarretia* sp. |
|  |  |  | *Phlox* spp. (2) |
|  | Asterales | Asteraceae | *Blennosperma* sp. |
|  |  |  | *Cichorium* sp. |
|  |  |  | *Stephanomeria* sp. |
|  |  |  | *Taraxacum* sp. |
|  |  |  | *Tragopogon* sp. |
|  |  |  | *Wyethia* sp. |
|  | Escalloniales | Escalloniaceae | *Escallonia* sp. |
|  | Dipsacales | Caprifoliaceae | *Lonicera* sp. |
|  | Solanales | Convolvulaceae | *Calystegia* sp. |
|  |  | Solanaceae | *Capsicum* sp. |
|  |  |  | *Nicotiana* sp. |
|  |  |  | *Petunia* sp. |
|  | Lamiales | Oleaceae | *Forsythia* sp. |
|  |  |  | *Ligustrum* sp. |
|  |  |  | *Syringa* sp. |
|  |  | Plantaginaceae | *Antirrhinum* sp. |
|  |  |  | *Collinsia* sp. |
|  | Gentianales | Apocynaceae | *Mandevilla* sp. |
|  |  |  | *Trachelospermum*sp. |
|  |  |  | *Asclepias* spp. (3) |

**Supplementary Table S4.** Flowering plant species with floral nectar that contain known fluorophores or related compounds, specifically chlorogenic acids, genistein-related isoflavones or glycosides, and aesculetin. Species with known UV-fluorescent floral nectar are indicated with an asterisk. Of note, Palmer-Young *et al.* [5] referred to all phenylpropenoid derivatives of quinic acid as chlorogenic acids.

| Group | Order | Family | Species | Chemical class | Chemical compound | References |
| --- | --- | --- | --- | --- | --- | --- |
| Monocots | Pandanales | Stemonaceae | *Stemona tuberosa* | Hydroxycinnamate derivative | Chlorogenic acid | [4] |
| Eudicots | Ranunculales | Papaveraceae | *Dicentra eximia* | Hydroxycinnamate derivative | Chlorogenic acid | [5] |
|  | Fabales | Fabaceae | *Robinia pseudoacacia** | Hydroxycinnamate derivative | Chlorogenic acid | [6] |
|  |  |  |  | Isoflavone | Genistein | [6] |
|  |  |  | *Trifolium pratense* | Isoflavone glycoside | Genistein derivative | [5] |
|  |  |  |  | Isoflavone acylglycoside | Genistein derivative | [5] |
|  | Rosales | Rosaceae | *Malus domestica* | Hydroxycinnamate derivative | Chlorogenic acid | [5] |
|  |  |  | *Prunus avium* | Hydroxycinnamate derivative | Chlorogenic acid | [5] |
|  |  |  | *Pyrus* spp. | Hydroxycinnamate derivative | Chlorogenic acid | [7] |
|  |  |  |  | Hydroxycoumarin | Aesculetin | [7] |
|  | Cucurbitales | Cucurbitaceae | *Cucurbita pepo* | Hydroxycinnamate derivative | Chlorogenic acid | [5] |
|  | Sapindales | Rutaceae | *Citrus reticulata* | Hydroxycinnamate derivative | Chlorogenic acid | [8] |
|  |  |  |  | Isoflavone | Genistein | [8] |
|  |  |  | *Citrus tangerina* | Hydroxycinnamate derivative | Chlorogenic acid | [8] |
|  |  |  |  | Isoflavone | Genistein | [8] |
|  | Caryophyllales | Polygonaceae | *Fagopyrum esculentum** | Hydroxycinnamate derivative | Chlorogenic acid | [9] |
|  | Ericales | Ericaceae | *Rhododendron roseum* | Hydroxycinnamate derivative | Chlorogenic acid | [5] |
|  |  |  | *Vaccinium corymbosum* | Hydroxycinnamate derivative | Chlorogenic acid | [5] |
|  | Lamiales | Plantaginaceae | *Penstemon digitalis* | Hydroxycinnamate derivative | Chlorogenic acid | [5] |

**References**

1. Thorp, R. W., Briggs, D. L., Estes, J. R. & Erickson, E. H. Nectar fluorescence under ultraviolet irradiation. *Science* **189**, 476–478 (1975).
2. Roshchina, V. V., Melnikova, E. V., Mit’kovskaya L. V. & Karnaukhov, V. N. Microspectrofluorimetry for the study of intact plant secreting cells. *J. Gen. Biol.* **59**, 531–553 (1998).
3. Scogin, R. Reproductve phytochemistry of Bombacaceae: floral anthocyanins and nectar constituents. *Aliso: J. Syst. Florist. Bot.* **11**, 377–385 (1986).
4. Cai, X.-H. *et al.* Mystery revisited: is nocturnal colored nectar a nonadaptive floral trait? *Ecology* **103**, e3663 (2022).
5. Palmer-Young, E. C. *et al.* Chemistry of floral rewards: intra- and interspecific variability of nectar and pollen secondary metabolites across taxa. *Ecol. Monogr.* **89**, e01335 (2019).
6. Gismondi, A. *et al.* From *Robinia pseudoacacia* L. nectar to Acacia monofloral honey: biochemical changes and variation of biological properties. *J. Sci. Food Agric.* **98**, 4312–4322 (2018).
7. Fotirić Akšić, M. *et al*. The morpho-anatomy of nectaries and chemical composition of nectar in pear cultivars with different susceptibility to *Erwinia amlylovora*. *Horticulturae* **9**, 424 (2023).
8. Gao, Y. *et al.* Analysis of chemical composition of nectars and honeys from *Citrus* by extractive electrospray ionization high resolution mass spectrometry. *LWT* **131**, 109748 (2020).
9. Nešović, M. *et al.* Polyphenol profile of buckwheat honey, nectar and pollen. *R. Soc. Open Sci.* **7**, 201576 (2020).
